# The Electrically Evoked Compound Action Potential: From Laboratory to Clinic

**DOI:** 10.3389/fnins.2017.00339

**Published:** 2017-06-23

**Authors:** Shuman He, Holly F. B. Teagle, Craig A. Buchman

**Affiliations:** ^1^Center for Hearing Research, Boys Town National Research HospitalOmaha, NE, United States; ^2^Department of Otolaryngology—Head and Neck Surgery, University of North Carolina at Chapel HillChapel Hill, NC, United States; ^3^Department of Otolaryngology—Head and Neck Surgery, Washington UniversitySt. Louis, MO, United States

**Keywords:** electrically evoked compound action potential, stimulating paradigm, clinical application, auditory nerve, cochlear implant outcome

## Abstract

The electrically evoked compound action potential (eCAP) represents the synchronous firing of a population of electrically stimulated auditory nerve fibers. It can be directly recorded on a surgically exposed nerve trunk in animals or from an intra-cochlear electrode of a cochlear implant. In the past two decades, the eCAP has been widely recorded in both animals and clinical patient populations using different testing paradigms. This paper provides an overview of recording methodologies and response characteristics of the eCAP, as well as its potential applications in research and clinical situations. Relevant studies are reviewed and implications for clinicians are discussed.

## Introduction

The electrically evoked compound action potential (eCAP) represents a synchronized response generated by a group of electrically activated auditory nerve fibers. Current cochlear implants (CI) incorporate a “reverse” telemetry capability that allows near-field recordings of the eCAP using intra-cochlear electrodes. Compared with other electrophysiological measures, the eCAP offers several advantages that make it of great value to hearing scientists and audiologists. First, measuring the eCAP in CI patients does not require extra equipment, special software, or an external recording electrode other than the standard equipment for clinical programming. It can be done through the telemetry function implemented in the CI and the commercial software provided by the manufacture. Second, it requires minimal patient cooperation and is not affected by patient's arousal status, which is an important advantage for working with pediatric CI users. Additionally, it is known to be a stable measure overtime in typical CI recipients and therefore can be a reliable indicator of change.

Electrical stimuli delivered by the CI are first encoded by the auditory nerve, and subsequently transmitted to higher auditory neural structures. Theoretically, the ability of the auditory nerve to faithfully encode and process electrical stimuli should be important for CI outcomes. Results of several studies suggest that the physiological status (i.e., number and responsiveness of neurons) of the auditory nerve may be important for CI outcomes (e.g., Kim et al., 2010; Kirby and Middlebrooks, 2010, 2012; Garadat et al., 2012, 2013; Long et al., 2014; Pfingst et al., 2015a,b). The eCAP is a direct measurement of neural responses generated by auditory nerve fibers, which makes it feasible to exclusively evaluate the physiological status of the auditory nerve. Many studies have focused on evaluating the feasibility of using the eCAP to determine stimulus levels for individual electrodes in CI patients (e.g., Brown et al., 2000; Hughes et al., 2000; Thai-Van et al., 2001; Gordon et al., 2002, 2004; Eisen and Franck, 2004). Over the past 10 years, there has been a steady increase in the number of studies using the eCAP to assess different aspects of responsiveness of the auditory nerve and their associations with CI outcomes in both adult and pediatric CI users (e.g., Botros and Psarros, 2010; Hughes et al., 2012; Lee et al., 2012; He et al., 2016a). This article provides an overview of these studies, with an emphasis on several potential applications of the eCAP in research and clinical situations in human CI users.

## General overview

### Brief history

Even though the acoustically evoked compound action potential (CAP) has been widely used in basic and clinical studies for more than six decades (Goldstein and Kiang, 1958), the feasibility of measuring the eCAP in animals or human listeners was not established until late 1980s (van de Honert and Stypulkowski, 1986; Game et al., 1987; Miyamoto and Brown, 1987; Abbas and Brown, 1988). The delay is primarily due to the lack of technique for recognizing and minimizing contamination of stimulus artifact on the recorded response. In 1990, Brown et al. developed a forward-masking technique for measuring the eCAP from an intra-cochlear electrode in human CI patients (Brown et al., 1990). This technique can successfully minimize stimulus artifact and allow artifact-free eCAPs to be recorded. Telemetry function became commercially available for eCAP recording in 1998, when Cochlear™ Limited (Sydney, Australia) incorporated two-way telemetry in the Nucleus® CI24 CI (Neural Response Telemetry [NRT]). In 2001, Advanced Bionics (Valencia, California) followed by including telemetry capability in their devices (Neural Response Imaging [NRI]). MED-EL's (Innsbruck, Austria) version of telemetry (Auditory Response Telemetry [ART]) was commercially approved in the United States in 2007.

### eCAP morphology

**Figure 1:**
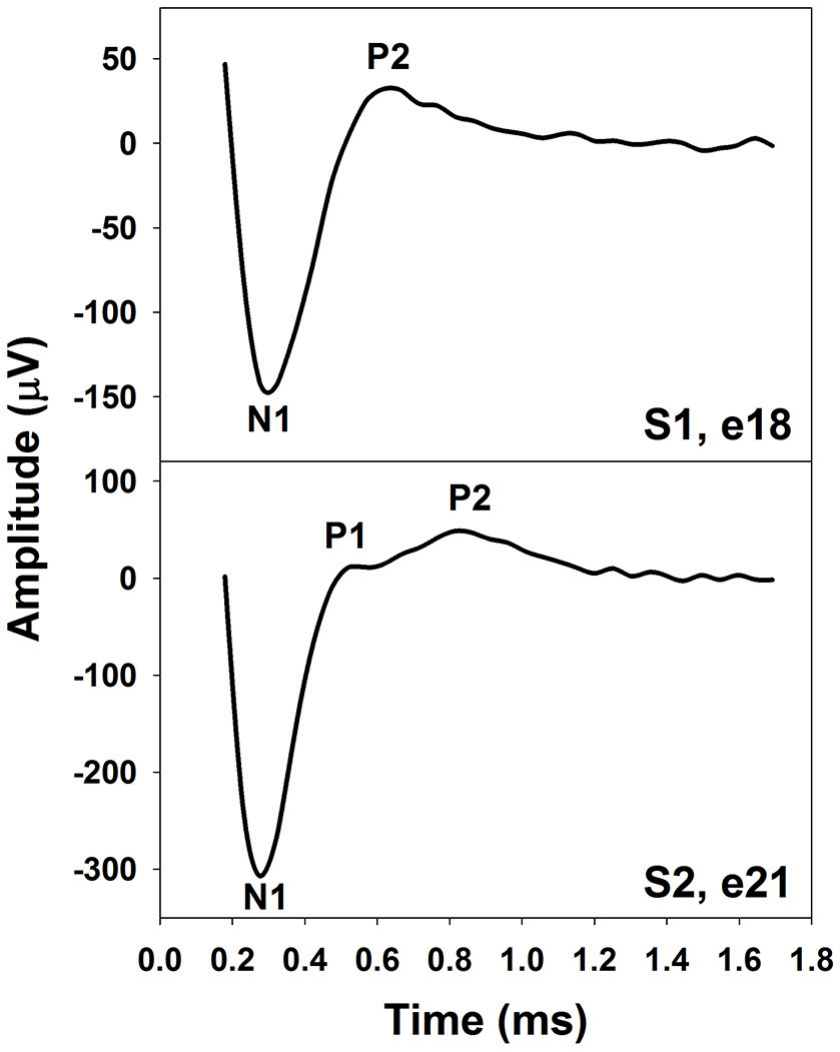
The eCAP with one **(Upper)** or two positive peaks **(Lower)**. These responses were recorded using intra-cochlear electrodes in pediatric Cochlear Nucleus CI users with prelingual deafness. Subject and electrode number are labeled in both panels.

The eCAP recorded using an intra-cochlear electrode in human CI users typically shows a biphasic morphology. The upper panel of Figure 1 shows an example of an eCAP recorded in one pediatric Cochlear 24RE CI user with prelingual deafness. The biphasic eCAP consists of one negative peak (N1) within a time window of 0.2–0.4 ms after stimulus onset followed by a positive peak (P2) occurring around 0.6–0.8 ms (Brown and Abbas, 1990; Brown et al., 1990, 1998; Abbas et al., 1999). This single-peak eCAP accounts for more than 80% of all measurable eCAPs (Lai and Dillier, 2000; Cafarelli Dees et al., 2005; Miller et al., 2008b).

In addition to the single-peak response, eCAPs with two positive peaks (P1 and P2) have been observed (Stypulkowski and van den Honert, 1984; Lai and Dillier, 2000; van de Heyning et al., 2016). This type of response has been referred to as a double-peak or a Type II nerve response (Lai and Dillier, 2000). For this type of eCAP response, the P1 typically occurs around 0.4–0.5 ms and the P2 typically occurs around 0.6–0.7 ms (Lai and Dillier, 2000; van de Heyning et al., 2016). The incidence of the Type II response is around 10–20% (Lai and Dillier, 2000; van de Heyning et al., 2016). The lower panel of Figure 1 shows an example of a Type II response measured in a prelingually deaf child with a Cochlear N5 CI. Based on results recorded in cats, Stypulkowski and van den Honert (1984) proposed that the P1 and the P2 peak arise from action potentials generated by the axonal and the dendritic processes, respectively. Latency differences between these two peaks might reflect the time of spike propagation along the dendrite and across the spiral ganglion cell body. This “two-component” hypothesis is supported by simulation results of a mathematical model including a liner combination of responses generated by axons and dendrites (Lai and Dillier, 2000).

The eCAP amplitude can be as large as 1–2 mV. Due to its large amplitude, the eCAP is relatively resistant to contamination of myogenic activity. In addition, due to its peripheral neural origin, the eCAP is not affected by maturation of the central auditory system. As a result, morphological characteristics of eCAPs recorded in adult and pediatric CI users are similar (e.g., Brown et al., 1990; Eisen and Franck, 2004; Gordon et al., 2004) and show little or no change as the duration of CI use increases (Brown et al., 2010). Nevertheless, amplitude and peak latency of the eCAP recorded in human CI users are affected by extrinsic factors, including stimulation level, intra-cochlear test electrode location, the separation between stimulating and recording electrodes, stimulus polarity, etc. For example, eCAP amplitude increases as the stimulation level increases. The speed of the increase can be quantified by the slope of an eCAP input-output (I/O) function. In addition, eCAPs recoded at the apical electrodes tend to have larger amplitudes than those recorded at the basal electrodes at an equal stimulus or loudness level (e.g., Frijns et al., 2002; Polak et al., 2004; Brill et al., 2009; van de Heyning et al., 2016; Tejani et al., 2017). Potential factors accounting for the increase in eCAP amplitude toward the apical region include better neural survival and shorter distance between the test electrode and the stimulated neural structure at the apex. As the separation between stimulating and recoding electrodes increases, the eCAP latency may decrease (Finley et al., 2013; Kashio et al., 2016) due to potential changes in the site of action potential initiation (Kashio et al., 2016). Finally, the eCAP evoked by the anodic-leading biphasic pulse has a larger amplitude and shorter latency than that evoked by the cathodic-leading biphasic pulse at the same stimulus level (e.g., Macherey et al., 2006, 2008; Undurraga et al., 2010, 2012; Baudhuin et al., 2016). The proposed underlying neurophysiological mechanism is that auditory nerve fibers with degenerated or unmyelinated peripheral processes are more sensitive to anodic than to cathodic stimulation (Rattay, 1999; Rattay et al., 2001; Macherey et al., 2008; Undurraga et al., 2010, 2012). Details of this mechanism are described later in the Polarity Sensitivity section.

### Artifact rejection methods

Ideally, the eCAP is recorded from the same intra-cochlear electrode that delivers electrical stimulus. However, this is not feasible due to residual decaying charges of the electrical stimulus (i.e., artifact). This artifact is often large enough to saturate the recording amplifier. Once the amplifier is saturated, no response can be recorded before it recovers, which is problematic for measuring the eCAP due to its short latency. In practice, the stimulating and recording electrodes used for intra-cochlear eCAP measures are typically separated by one or two electrodes. Unfortunately, the physical separation between the stimulating and recording electrodes cannot completely eliminate the distortion introduced by the stimulus artifact. Additional artifact reduction techniques are typically needed for measuring the artifact-free eCAP. Each method is described as follows.

**Figure 2:**
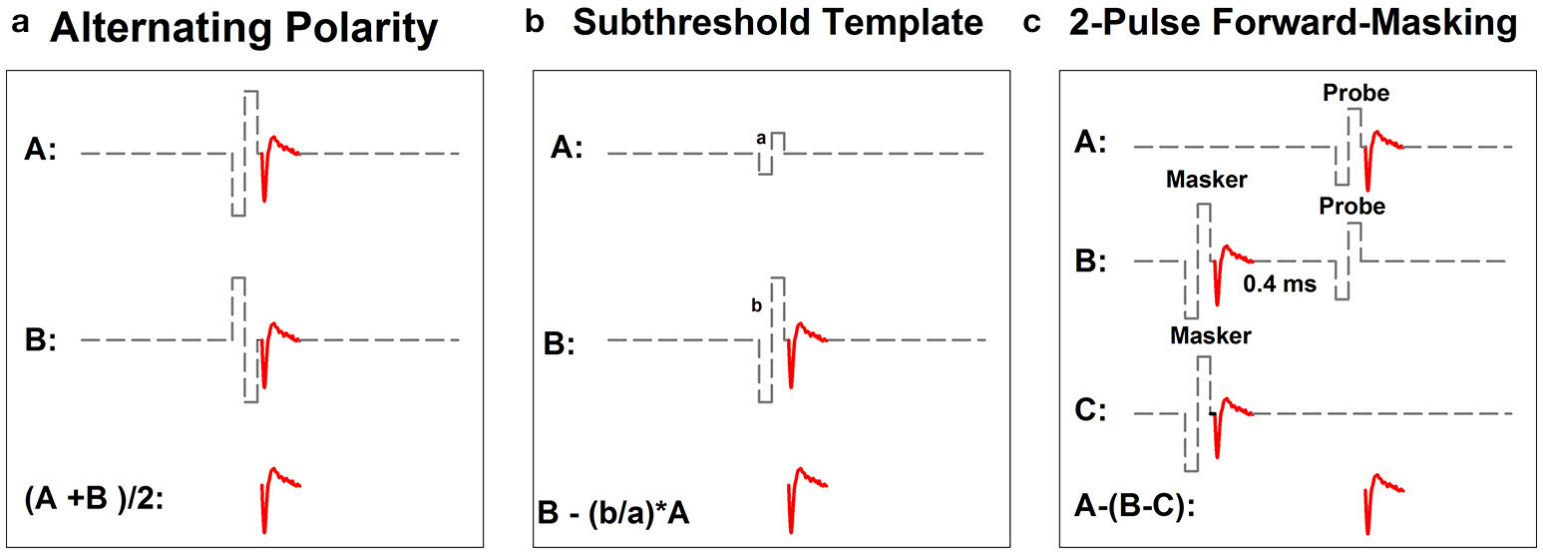
Schematic illustration of three methods for minimizing contamination of stimulus artifact in eCAP recordings: alternating-polarity **(a)**, subthreshold template **(b)** and 2-pulse forward masking paradigm **(c)**. Gray dashed lines and red solid lines indicate biphasic electrical pulses and eCAP responses, respectively.

Figure 2 shows schematic illustrations of the three most commonly used artifact reduction techniques for measuring the intra-cochlear eCAP: alternating polarity (Figure 2a), subthreshold template subtraction (Figure 2b), and two-pulse forward-masking paradigm (Figure 2c). Alternating polarity method is used in Advanced Bionics' NRI and MED-EL's ART programs. All three methods are offered as options in Cochlear's NRT software.

In the alternating polarity method, responses (including the artifact and the eCAP) evoked by the cathodic-leading (trace A) and the anodic-leading (trace B) biphasic pulse are recorded. The polarity of the stimulus artifact in these two traces is reversed. In contrast, the polarity of the eCAP remains the same. The stimulus artifact is eliminated or minimized and the eCAP is derived by averaging the responses of both polarities (i.e., (A+B)/2). While simple in theory, the success of this method depends on the underlying assumption that eCAPs evoked by cathodic- or anodic-leading biphasic pulses are identical. Unfortunately, this assumption is not valid. Results of recent studies have shown that human auditory nerve fibers are more sensitive to anodic-leading than cathodic-leading biphasic pulses (e.g., Macherey et al., 2006, 2008; Undurraga et al., 2010). As a result, eCAPs in response to stimuli with reversed polarities differ in amplitude and latency (Frijns et al., 2002; Macherey et al., 2006, 2008; Undurraga et al., 2010; Baudhuin et al., 2016). Therefore, using the alternating polarity artifact reduction method may result in distorted eCAP responses (Frijns et al., 2002; Baudhuin et al., 2016).

The subthreshold template subtraction method (Figure 2b) was first proposed by Miller et al. (1998) in their animal studies. In this method, a response evoked by a biphasic pulse that is below neural threshold is recorded (trace A). This trace contains only stimulus artifact, which serves as the template. Trace B contains the stimulus artifact and the eCAP evoked by a supra-threshold biphasic pulse. The template is then scaled up to match the magnitude of stimulus artifact in trace B. The eCAP is derived by subtracting the scaled template from trace B. Successfully implementing this paradigm requires a precise and unerring recording system with a linear recording amplifier, a low level of ambient noise, and the capability of accurately sampling stimulus artifact. As a result, the subthreshold template subtraction method is used less frequently than the other two methods in studies with human CI users.

The two-pulse forward masking paradigm (Figure 2c) takes advantage of refractory properties of the auditory nerve (Brown et al., 1990). In this paradigm, responses are recorded in four stimulation conditions. In the first condition (trace A), a response evoked by a single biphasic pulse (i.e., the probe) is recorded. This response includes the stimulus artifact and the eCAP evoked by the probe. In the second condition (trace B), two biphasic pulses are presented sequentially with a relatively short inter-pulse interval. The first pulse (i.e., the masker) is typically higher in stimulation level than the second pulse (i.e., the probe). When the masker-probe-interval (MPI) is sufficiently short (~350–400 μs), the response to the masker is assumed to leave the nerve in a refractory state such that it is unable to generate a neural response to the probe. Therefore, the trace recorded in this condition includes artifacts evoked by the masker and the probe and the eCAP evoked by the masker. In the third condition (trace C), only the masker is presented and the recorded response includes the artifact and the eCAP evoked by the masker. The fourth condition (not shown in this illustration) is used to control for power-up artifact of the recording system. The eCAP elicited by the probe can be derived by subtracting artifact evoked by the probe (i.e., B-C) from the response evoked by the probe alone (i.e., A-(B-C)). The success of this paradigm depends on the absence of neural response evoked by the probe in trace B. Unintended neural response to the probe will be evoked if the masking effect induced by the masker is insufficient in cases where the MPI is too long/short or the level of the masker is too low.

## Applications

Potential clinical application of the eCAP has been extensively studied. Despite that many studies were done in patients with Cochlear Nucleus devices, general knowledge gained from these studies applies to any CI users. Much of the early literature on this topic focused on using the eCAP to determine program levels for individual CI electrodes (e.g., Brown et al., 1998, 2000; Abbas et al., 1999; Hughes et al., 2000; Franck and Norton, 2001; Gordon et al., 2002, 2004; Smoorenburg et al., 2002; Eisen and Franck, 2004; Thai-Van et al., 2004; McKay et al., 2005, 2013; Potts et al., 2007). Accumulating evidence suggests that the status of the auditory nerve may be important for CI outcomes (e.g., Garadat et al., 2012, 2013; Kirby and Middlebrooks, 2012; Pfingst et al., 2015a,b). In addition, eCAPs have been shown to be sensitive to electrode placement and the health status of auditory nerve fibers near the recording electrode (Shepherd et al., 1993; Miller et al., 2008a). Therefore, recent literature has been focusing on using the eCAP to evaluate neural survival (e.g., Botros and Psarros, 2010; Kim et al., 2010; Pfingst et al., 2015a) and spectral and temporal encoding of electrical stimulus at the level of the auditory nerve and their associations with auditory perception in CI users (e.g., Hughes and Abbas, 2006; Hughes and Stille, 2008; Hughes et al., 2012; Snel-Bongers et al., 2012; Carlyon and Deeks, 2015; Scheperle and Abbas, 2015a,b; DeVries et al., 2016; He et al., 2016a; Tejani et al., 2017). The following section summarizes studies investigating potential applications of the eCAP in each of these areas.

### Clinical programming

Clinical programming of a CI speech processor requires estimations of the lowest level that patients can detect (T level) and the upper limit of the level that patients determine to be comfortable (C or M level) for multiple stimulating electrodes. Optimal C level allows accessing loud sound without causing discomfort. Accurate T level has been shown to be critical for understanding low-level speech and speech presented in noise (e.g., Skinner et al., 1997, 1999, 2002; James et al., 2003; Firszt et al., 2004; Dawson et al., 2007; Holden et al., 2007, 2011; Spahr et al., 2007; Davidson et al., 2009; Baudhuin et al., 2012; van der Beek et al., 2015). Measuring T and C levels for multiple stimulating electrodes is time consuming and requires a significant amount of attention and effort to accomplish. Further complicating programming efforts is the fact that some CI users have limited abilities to provide reliable behavioral responses due to their young age or other comorbidities. Having objective tools for determining stimulus levels can potentially accelerate the programming process and be especially useful for managing patients who cannot perform behavioral tasks.

The feasibility of using the eCAP evoked by a single biphasic pulse to estimate T and C levels has been extensively evaluated in both adult and pediatric CI users (Brown et al., 1998, 2000; Abbas et al., 1999; Hughes et al., 2000; Franck and Norton, 2001; Gordon et al., 2002, 2004; Smoorenburg et al., 2002; Eisen and Franck, 2004; Thai-Van et al., 2004; Han et al., 2005; McKay et al., 2005; Potts et al., 2007; Wolfe and Kasulis, 2008; Holstad et al., 2009; Jeon et al., 2010). Overall, results of these studies suggest that stimulus at the level of eCAP threshold is always audible to CI patients. However, there is only a weak to moderate correlation between eCAP thresholds and behavioral T or C levels in both adult and pediatric CI users. The reported correlation coefficients vary across studies. For the correlation between eCAP thresholds and T levels, the reported coefficients range from 0.5 to 0.9. For the correlation between eCAP thresholds and C levels, the reported coefficients range from 0.1 to 0.9. The correlation between eCAP thresholds and T and C levels appears to be stronger at the apical compared to the basal electrodes (Eisen and Franck, 2004; Wolfe and Kasulis, 2008). Even though the eCAP threshold typically falls between behavioral T and C levels, there are substantial variations among patients, as well as across CI electrodes within individual patients. It is common for the eCAP threshold to exceed C level, especially at high stimulation rates (Eisen and Franck, 2004; Han et al., 2005; Jeon et al., 2010).

It has been proposed that the difference in stimulus used for eCAP measures (a single pulse presented at 80 Hz or lower) and behavioral procedures [a train of pulses with pulse rates of 250 pulses per second (pps) or higher] could, at least partially, account for the lack of robust correlation between these two measures (McKay et al., 2005). Specifically, the eCAP to a single biphasic pulse is relatively independent of the history of prior neural activity and mainly reflects the inherent excitability of the electro-neural interface. In contrast, behavioral T and C levels measured using a train of pulses are affected by additional peripheral and central factors. For example, responsiveness of the auditory nerve to the pulse-train stimuli is affected by many neural response properties, including peri-stimulus neural refractoriness and adaptation, as well as recovery from refractoriness and adaptation induced by prior stimulation. In addition, auditory perception of a pulse train is affected by auditory temporal integration that is generally believed to occur at the central auditory system (Viemeister and Wakefield, 1991; McKay and McDermott, 1998). Therefore, several studies have tried to address this caveat by using similar stimuli for eCAP and behavioral measures. The correlation between eCAP threshold and behavioral T and C levels improves when low rate pulses (e.g., 80 Hz or lower) are used in both measures (Brown et al., 1996, 1998; Zimmerling and Hochmair, 2002). Nevertheless, substantial inter- and intra-subject variations in the relationship between these two measures still exist. McKay et al. (2013) explored the feasibility of using eCAP evoked by trains of biphasic pulses at different pulse rates to predict behavioral T and C levels in both adult and pediatric CI users. Unfortunately, their results revealed insufficient predictive power of eCAP measure for setting program levels for individual patients.

Several methods have been proposed for improving the correlation between eCAP threshold and behavioral T and C levels. For example, Brown et al. (2000) and Hughes et al. (2000) plotted eCAP thresholds as a function of the electrode location. This function was then shifted up and down based on the difference in stimulus level between eCAP threshold and behavioral T and C levels that was measured for one electrode. This method improves overall correlations between eCAP threshold and behavioral T and C levels in both adult and pediatric CI users. However, it does not work well for patients whose behavioral T and C levels vs. electrode contours are different from eCAP threshold vs. electrode contours (Miller et al., 2008a). In addition, programming maps created using this method do not lead to improved speech understanding in CI patients (Seyle and Brown, 2002; Smoorenburg et al., 2002). Combining eCAP threshold with the slope of the eCAP amplitude growth function has been shown to improve the correlation between eCAP threshold and behavioral C levels (Franck and Norton, 2001). The “tilt” of the eCAP threshold vs. electrode contour is more strongly correlated with behavioral T levels than the absolute eCAP threshold (Smoorenburg et al., 2002). Therefore, varying the “tilt/curvature” in addition to shifting the contour up and down has also been recommended (Smoorenburg et al., 2002). Nevertheless, it remains unknown whether these two additional methods would result in optimal program levels for CI outcomes.

In summary, eCAP threshold can provide information to clinicians about the function of the internal device and its interface with neural elements. In addition, it can provide an initial estimation of program levels, which is important for working with patients who cannot provide reliable behavioral responses. However, the poor predictive power of eCAP threshold for behavioral T and C levels prevents it from being used as a sole indicator for setting the program levels for individual patients. Accurate behavioral T and C levels are still warranted for optimal programming settings.

### Spectral resolution

Compared to normal hearing listeners, CI users are known to have impaired spectral resolution (e.g., Fu et al., 1998; Friesen et al., 2001; Loizou and Poroy, 2001; Henry and Turner, 2003; Jeon et al., 2015; Winn and Litovsky, 2015), and the severity of this deficits correlates with their speech perception capabilities (Fu et al., 1998; Friesen et al., 2001; Henry and Turner, 2003; Fu and Nogaki, 2004; Henry et al., 2005; Litvak et al., 2007; Won et al., 2007; Winn et al., 2016). The number of individual electrodes that provides perceptually distinct spectral information (i.e., effective spectral channels) has been proposed to be an important factor for spectral resolution in CI users (Friesen et al., 2001; Jones et al., 2013). The electrical current delivered by each CI electrode creates an electric field that stimulates the surrounding neural tissue. The electrical fields created by different electrodes typically overlap with each other, resulting in channel interactions wherein the same neural population is excited by more than one stimulating electrode. The lack of across-fiber independence reduces the number of “effective spectral channels” of a multichannel CI, which compromises speech perception in implanted patients (Zwolan et al., 1997; Throckmorton and Collins, 1999; Dawson et al., 2000; Henry et al., 2000; Friesen et al., 2001; Noble et al., 2013).

Electrophysiological measures of the eCAP can be used to assess channel interaction at the electrode-neural interface (i.e., spread of excitation or SOE). The amount of SOE can be estimated based on eCAP amplitudes measured at different spatial separations between the masker- and the probe-electrode (e.g., Miller et al., 2001; Cohen et al., 2003; Abbas et al., 2004; Eisen and Franck, 2005; Hughes and Abbas, 2006; Hughes, 2008; Hughes and Stille, 2008; Hughes and Goulson, 2011; Snel-Bongers et al., 2012; Undurraga et al., 2012; van der Beek et al., 2012; Won et al., 2014; Scheperle and Abbas, 2015a,b). To evaluate SOE, the eCAP can be measured using either a two-pulse forward-masking/channel-interaction paradigm (e.g., Eisen and Franck, 2005; Hughes and Abbas, 2006; Hughes, 2008; Hughes and Stille, 2008; Hughes and Goulson, 2011; Snel-Bongers et al., 2012; Undurraga et al., 2012; van der Beek et al., 2012; Won et al., 2014) or a modified template subtraction method (Cohen et al., 2003; Abbas et al., 2004). In both methods, the probe-electrode is typically fixed and the masker-electrode is varied across the electrode array.

**Figure 3:**
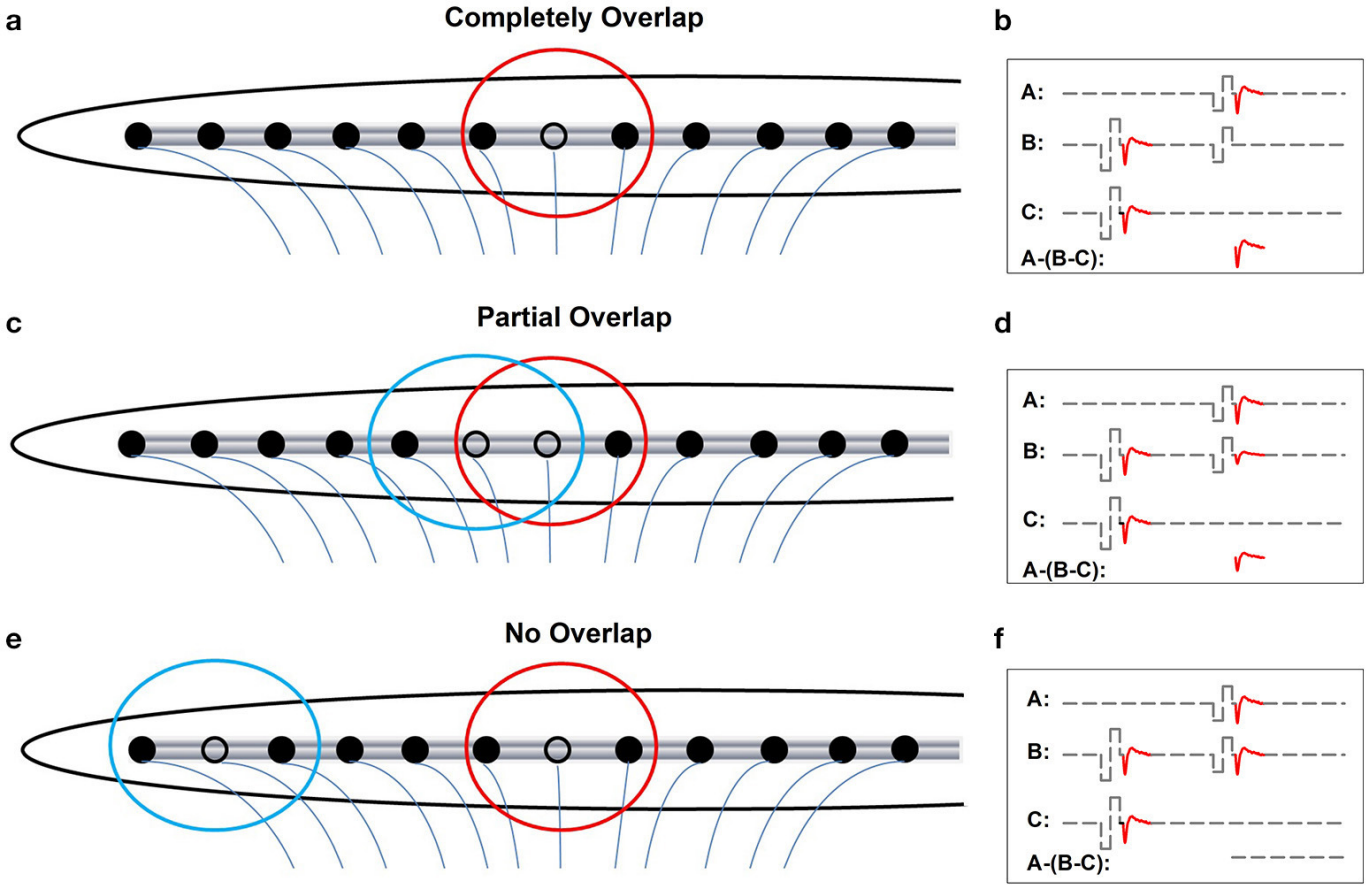
Schematic illustration of effects of increasing distance between the masker- and the probe-electrode on recorded eCAP response. Blue and red circles indicate electrode field evoked by the masker and the probe pulse, respectively. **(a, b)** Illustrate the condition where the masker pulse and the probe pulse are delivered to the same electrode. **(c, d)** Illustrate cases where the masker- and the probe-electrode are close to each other, which yields partially overlapped electrode fields. **(e, f)** Illustrate conditions where there is a large separation in distance between the masker- and the probe-electrode, which results in two separated electrical fields.

Figures 3a,c,e show schematic illustrations of relationships between electrode-spatial separations and neural populations activated by the probe and the masker. Figures 3b,d,f show schematic illustrations of measured eCAPs in these stimulation conditions using the two-pulse forward-masking/channel-interaction paradigm. In Figure 3a, the masker and the probe are presented on the same stimulating electrode (black open circle). Electrical fields (red circle) created by these two pulses are completely overlapped, which leads to activating only one group of neurons. Coupled with a short masker-probe-interval (MPI), all neurons that respond to the probe (trace A) are set into the refractory stage by the masker, which results in no neural response evoked by the probe in trace B in Figure 3b. The derived eCAP (the bottom trace of panel [b]) has the largest amplitude among all conditions shown in Figure 3. Figure 3c, the masker and the probe are presented on two adjacent electrodes. The electrical field created by the masker (blue circle) partially overlaps with that created by the probe (red circle), which leaves a subgroup of neurons that are unaffected by the masker pulse and thus can be activated by the probe. Consequently, trace B of Figure 3d contains a small response generated by these neurons in response to the probe, leading to a small eCAP in the subtracted trace (A-[B-C]). In Figure 3e, the masker and the probe are presented to two electrodes that are spatially separated by a large distance. There is no overlap between electrical fields created by these two pulses. The neural population that responds to the probe is unaffected by the masker. As a result, the eCAP evoked by the probe is recorded in trace B of Figure 3f. No eCAP is obtained after the subtraction (bottom trace of Figure 3f). Therefore, eCAP amplitudes as a function of spatial separations between the masker- and the probe-electrode provide an indication of the degree of overlap in the stimulated neural populations. This can be use used to quantify channel interaction occurring at the peripheral auditory system.

Compared with the two-pulse forward-masking/channel interaction paradigm, the modified template subtraction method is less commonly used and is not implemented in current telemetry capabilities by any CI manufacture. Details of this method have been described in Abbas et al. (2004). Briefly, the artifact evoked by the probe pulse is derived by subtracting trace C from trace B in cases where the masker and the probe are presented on the same electrode (Figure 3b), which serves as the “artifact template.” Contamination of stimulus artifact on recorded eCAPs is then removed or minimized by subtracting this “artifact template” from subtracted trace (B-C) recorded when the masker is presented on different electrodes. The template subtraction method results in the smallest eCAP when the neuronal overlap is greatest and vice versa.

**Figure 4:**
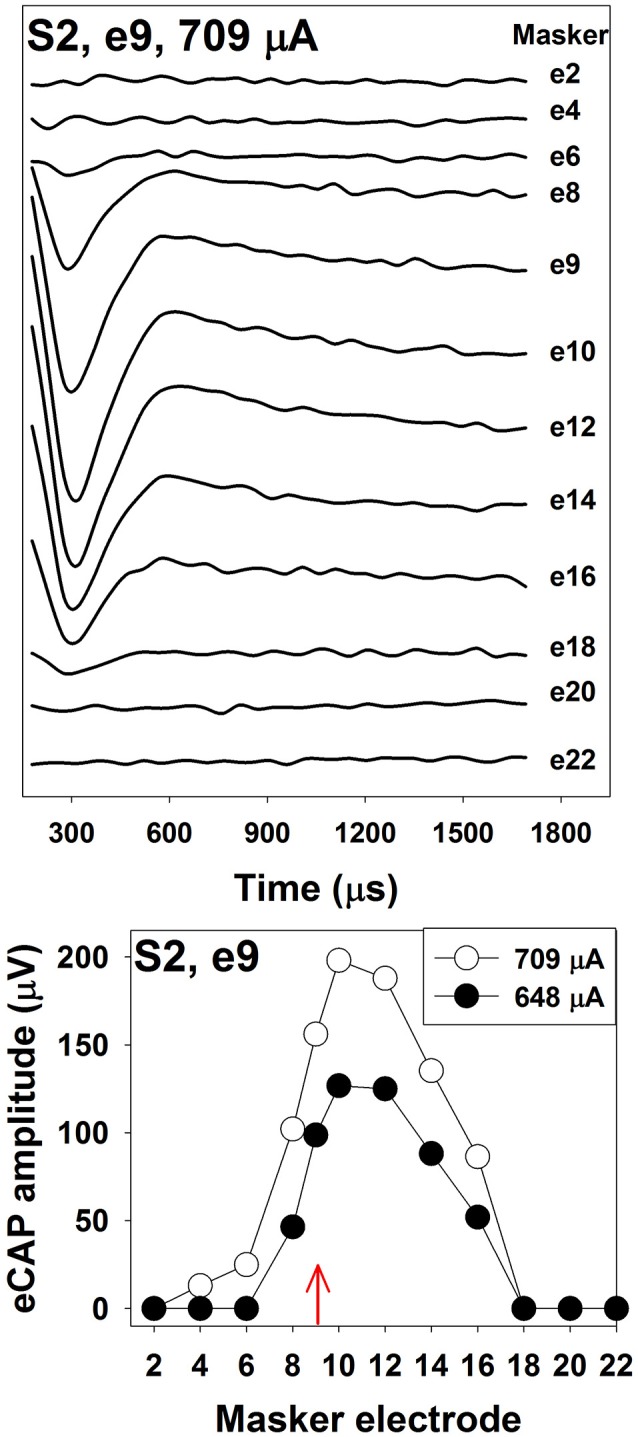
The top panel shows eCAP response series used to generate a channel-interaction function in S2. Each waveform is the derived eCAP measured for a probe pulse presented to electrode 9 at 709 μA. Masker electrode number is labeled for each trace. The bottom panel shows channel-interaction functions measured at 709 μA (open symbols) and 648 μA (filled symbols) in for the probe electrode 9 (red arrow) in S2.

The top panel of Figure 4 shows an example of one series of eCAP waveforms measured using the two-pulse forward-masking/channel-interaction paradigm in one pediatric Cochlear N5 CI user. The probe-electrode was fixed at electrode 9, and the masker-electrode location was systematically moved from electrode 2 to electrode 22. It is apparent that smaller spatial separations between the probe- and the masker- electrode result in larger eCAPs. The bottom panel shows eCAP amplitudes plotted as a function of masker-electrode locations (i.e., SOE function) measured at two stimulus levels. The function measured at 709 μA (open circles) is wider than that measured at 648 μA (solid circles). For this subject, the functions measured at both levels are asymmetrical, with more spread of neural excitation occurring at more apical masker electrodes. This asymmetry in excitation pattern is consistent with results reported in previous studies (Cohen et al., 2003; Abbas et al., 2004; Hughes and Stille, 2008; Hughes and Goulson, 2011; Scheperle and Abbas, 2015a,b). SOE functions vary in the overall amplitude, the width, and the shape among patients, as well as across electrode locations within individual CI users. Factors accounting for these variations include the stimulus level, the degree and pattern of neural survival, the electrode position relative to the stimulable neurons, the orientation of the electrodes and the resulting electrical field, and the impedance pathway for electrical current spread. To quantitatively compare the eCAP SOE function, eCAP amplitudes are typically normalized to the amplitude of the eCAP measured when the masker and the probe are presented on the same electrode.

Studies evaluating the association between eCAP SOE function, electrode pitch ranking and speech perception reveal mixed results. While most of these studies found no association between results of eCAP and behavioral measures (Cohen et al., 2003; Hughes and Abbas, 2006; Snel-Bongers et al., 2012; van der Beek et al., 2012), other studies reported that eCAP SOE functions were associated with electrode pitch ranking (Hughes, 2008) and speech perception in CI users (Won et al., 2014; Scheperle and Abbas, 2015a,b). Differences in the methodology used in these studies might account for the discrepancy in their results. For example, Hughes and Abbas (2006) measured the width of the eCAP SOE function at 75% of the normalized amplitude, and assessed its association with electrode pitch ranking ability and speech perception performance in CI users. Their results revealed no association among results of these measures. However, Hughes (2008) re-analyzed the same set of data by using the eCAP channel-separation index (CSI) to quantify SOE functions. Results showed a significant correlation between the eCAP SOE function and electrode pitch ranking ability, with less overlap of eCAP SOE functions associated with greater accuracy of electrode pitch ranking performance. Compared with the eCAP SOE width, the CSI is more sensitive to differences in locations and overall shapes of eCAP SOE functions. In addition, it provides a way for quantifying non-overlapped SOE functions. Therefore, it has been used in many recent studies (e.g., Abbas and Brown, 2015; Scheperle and Abbas, 2015a,b). For details of CSI calculation, please see Hughes (2008). The number of electrode locations tested may be another important factor to consider (Scheperle and Abbas, 2015b). Measuring the eCAP SOE function at few stimulating electrode locations may not capture the likely variability of SOE along the cochlea, which might partially account for the lack of correlation between eCAP SOE functions and speech perception reported in some studies (Cohen et al., 2003; van der Beek et al., 2012).

In summary, electrophysiological measures of the eCAP can be used to assess SOE pattern occurring at the electrode-neural interface. The CSI is a better parameter than the function width for quantifying the eCAP SOE function. Even though earlier literature showed no association between eCAP SOE function and behavioral measures of pitch ranking or speech perception, recent studies using the improved quantification method and more stimulating electrodes along the cochlea reported significant correlations among these measures. Nevertheless, the eCAP is generated by the auditory nerve. It does not provide information of auditory processing at the central auditory system that is important for speech perception. Scheperle and Abbas (2015a) found that eCAP SOE functions could only account for part of the variance observed in neural encoding of spectral information at the central auditory system. Therefore, the eCAP SOE function should not be used as the sole objective measure for predicting speech perception or electrode discrimination in CI users. However, this measure may provide useful information about channel interaction occurring at the electrode-neural interface, which leaves the possibility for new applications. For example, it can potentially be used to guard against tip fold-over electrode array during surgery. Further studies are warranted to test this speculation.

### Temporal responsiveness

Temporal information is important for speech perception in CI users, as minimal spectral cues are available to these patients. Temporal cues, especially rapid spectral and amplitude changes or acoustic onsets, are represented in the discharge patterns of the auditory nerve (Delgutte, 1980; Delgutte and Kiang, 1984). Evidence from recent studies suggests that temporal responsiveness of the auditory nerve plays an important role in encoding speech envelope cues (e.g., Kirby and Middlebrooks, 2012; Tejani et al., 2017). By using different stimulation paradigms, results of eCAP measures can provide information about many aspects of temporal response properties of the auditory nerve, including refractory recovery, neural adaptation, adaptation recovery, capability of encoding of amplitude modulation cues, etc. This section describes these eCAP stimulation paradigms and reviews related studies in human CI users.

#### Refractoriness and recovery

Refractoriness refers to a status in which neurons are incapable of generating an action potential immediately after a previous stimulation. It is a fundamental temporal property of the auditory nerve that enhances spike timing precision (Avissar et al., 2013). The time during which an action potential cannot be generated regardless of the magnitude of the stimulus is defined as the absolute refractory period (ARP). The ARP is followed by a relative refractory period (RRP) during which time the neuron can be activated by a strong stimulus. It has been shown that refractory properties have a significant effect on neural encoding of electrical pulse trains delivered by the CI at the level of the auditory nerve (Wilson et al., 1997).

**Figure 5:**
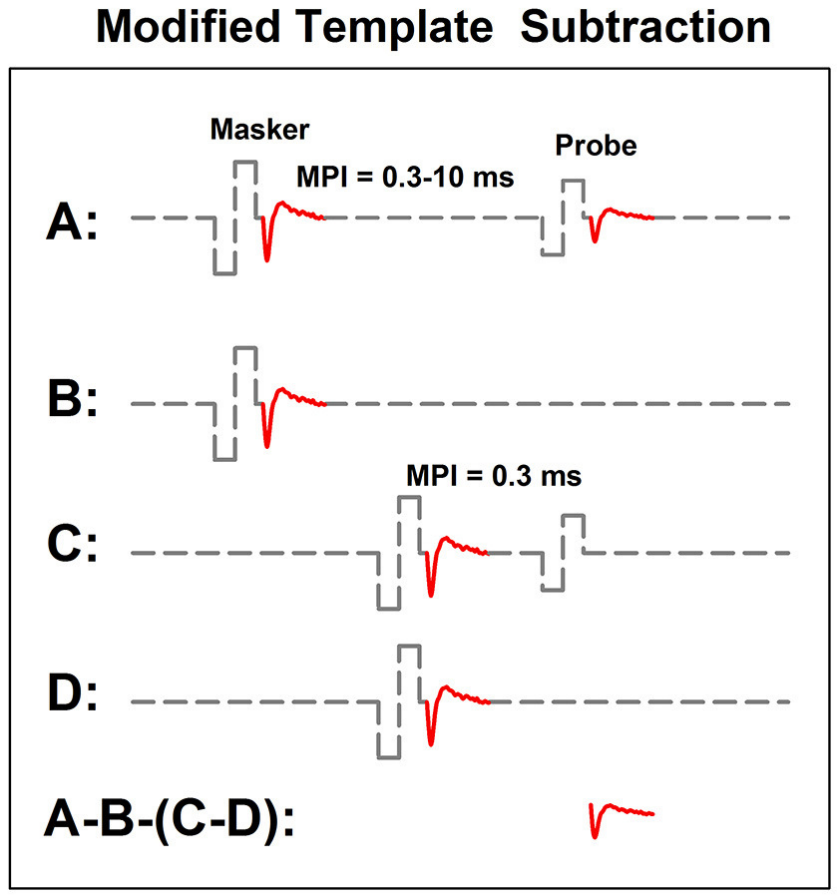
A schematic illustration of the modified template subtraction method for measuring the eCAP refractory recovery function. Gray dashed lines and red solid lines indicate biphasic electrical pulses and eCAP responses, respectively.

In human CI users, the ARP and the RRP can be estimated based on the eCAP refractory recovery function (RRF). The eCAP RRF is typically measured with two biphasic, charge-balanced, electrical pulses using a modified template subtraction method (Miller et al., 2000). A schematic illustration of this method is shown in Figure 5. In this paradigm, traces evoked by two masker-probe pairs are measured. The masker-probe-interval (MPI) of the first masker-probe pair systematically varies from 300 to 10,000 μs (trace A). As the MPI increases, the auditory nerve gradually recovers from the refractoriness induced by the masker, which results in larger eCAPs at longer MPIs in trace A. Subtracting trace “B” from trace “A” (i.e., A-B) yields the artifact and the eCAP evoked by the probe. The MPI of the second masker-probe pair is typically around 300 μs, which minimizes the neural response evoked by the probe (trace C) (Morsnowski et al., 2006). Subtracting trace “D” from trace “C” (i.e., C-D) results in the artifact evoked by the probe. The difference between these two derived traces (i.e., A-B-[C-D]) is the eCAP evoked by the first probe. The eCAP RRF is obtained by plotting (normalized) eCAP amplitudes as a function of MPIs.

**Figure 6:**
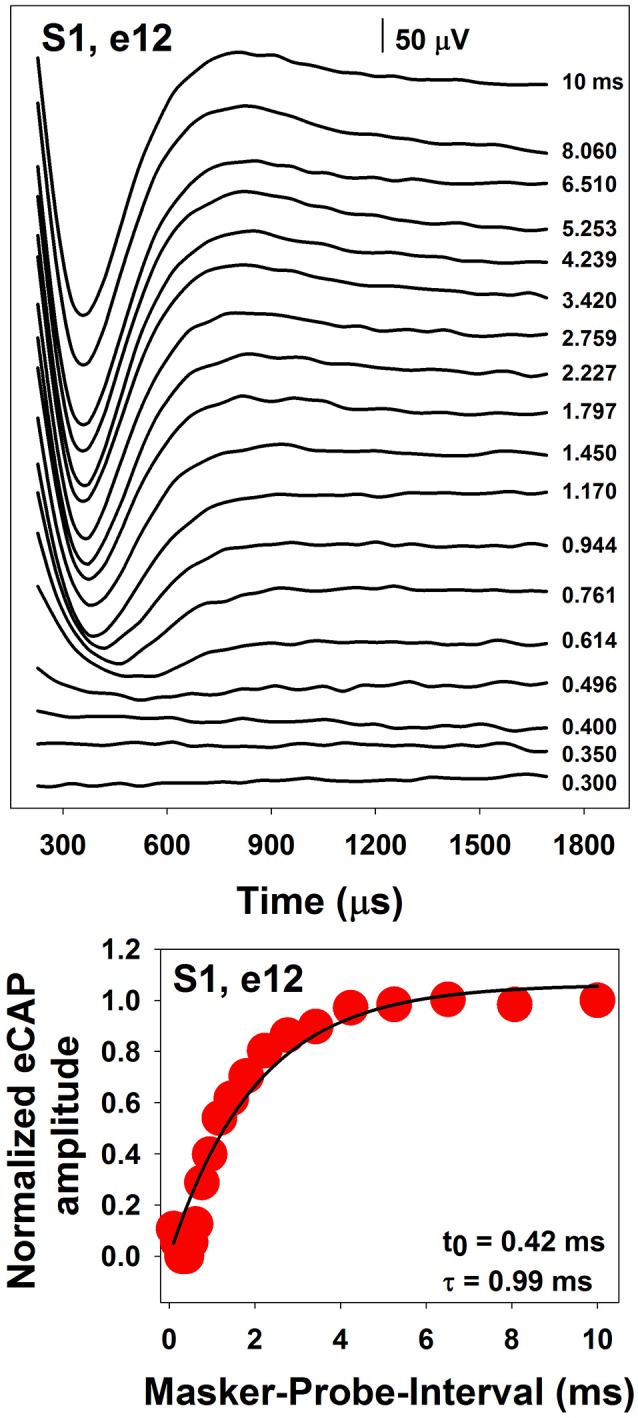
A series of eCAP waveforms **(Top)** and the derived eCAP refractory recovery function **(Bottom)** measured at electrode 12 in S1.

The top panel of Figure 6 shows a series of eCAP waveforms measured at different MPIs for electrode 12 in one pediatric CI user. MPIs used to measure these responses are labeled for these traces. These data clearly show that the eCAP becomes larger as the MPI increases. In this case, the eCAP amplitude was normalized to the amplitude of the eCAP measured at the MPI of 10 ms. The eCAP RRF was obtained by plotting the normalized eCAP amplitude (red symbol) as a function of MPIs, which is shown in the bottom panel of Figure 6. The eCAP RRF is typically modeled by an exponential decay function (e.g., Morsnowski et al., 2006; Botros and Psarros, 2010; Fulmer et al., 2011; He et al., 2017) of the form

(1) $$\begin{array}{r} eCAP_{N}=\text{ A}\left[1-{\text{e}}^{\frac{-\left(\text{MPI}-{\text{t}}_{0}\right)}{\tau }}\right], \end{array}$$

where eCAP_N_ represents the normalized eCAP amplitude, t_0_ is aligned with the ARP, τ is a measure of the speed of recovery from relative refractoriness (i.e., the RRP), and A represents the maximum eCAP amplitude evoked by the probe after a sufficiently long MPI. The line in the bottom panel of Figure 6 shows results of data fitting using this exponential decay function. Estimated t_0_ and τ are shown in the low right corner of this panel.

The speed of recovery from refractoriness is affected by stimulus level, with faster recovery at higher levels (Finley et al., 1997; Pesch et al., 2005). Medians/means of the ARP and the RRP measured at C level in “typical” CI users range from around 276 to 645 μs and from around 600 to 1350 μs, respectively (Pesch et al., 2005; Morsnowski et al., 2006; Hughes et al., 2012; Wiemes et al., 2016). Refractoriness measured for virtual vs. physical channels are comparable (Hughes and Goulson, 2011). Several studies have investigated refractory properties of the auditory nerve in some special patient populations, including children with auditory neuropathy spectrum disorder (ANSD) (Fulmer et al., 2011), elderly CI users (Lee et al., 2012), and children with cochlea nerve deficiency (CND) (He et al., 2017). Results of these studies showed that children with ANSD had similar refractory recovery time constants compared with children with typical sensorineural hearing loss (SNHL) (Fulmer et al., 2011). There is no association between refractory recovery time constants and chronological age (Lee et al., 2012). However, the RRP tends to prolong in patients with longer duration of hearing loss (Botros and Psarros, 2010; Lee et al., 2012). Compared with implanted children with normal-size auditory nerves, implanted children with CND have prolonged ARPs but similar RRPs (He et al., 2017).

Studies that investigated potential clinical applications of the eCAP RRF in optimizing programming rates and predicting CI outcomes reported inconsistent results (Brown et al., 1990; Abbas and Brown, 1991; Gantz et al., 1994; Kiefer et al., 2001; Shpak et al., 2004; Shpak, 2005; Fulmer et al., 2011; Lee et al., 2012). Shpak et al. (2004) reported a positive correlation between refractory recovery time constants and preferred programming rates. This finding was not replicated in a subsequent study by the same investigators (Shpak, 2005). Faster recovery from refractoriness has been reported to correlate with better speech perception scores in some studies (Brown et al., 1990; Kiefer et al., 2001; Fulmer et al., 2011). However, this association is not observed in other studies (Finley et al., 1997; Turner et al., 2002; Battmer et al., 2005; Lee et al., 2012). Factors accounting for these inconsistencies are unclear. One possibility is that the eCAP RRF may be affected by factors other than temporal responsiveness of the auditory nerve. For example, it has been proposed that refractory recovery time constants are affected by the size of neuron population. Specifically, prolonged ARP has been shown to be associated with reduced auditory nerve fiber density in rats (Shepherd et al., 2004). These results are consistent with prolonged ARPs estimated in children with CND (He et al., 2017). Based on simulation results of a computational model, Botros and Psarros (2010) proposed that longer RRPs were associated with better neural survival in CI patients. However, this theory is not supported by the relatively normal RRPs measured in children with CND who presumably have reduced number of neurons (He et al., 2017). Other factors, like difference in stimulation mode (bipolar vs. monopolar) and sample size, might also attribute to the inconsistent findings among these studies.

In summary, the ARP and the RRP of the electrically-stimulated auditory nerve can be estimated based on the eCAP RRF. To date, potential clinical application of the eCAP RRF is unclear due to limited research findings. Further studies with large sample sizes are warranted.

#### Neural adaptation and adaptation recovery

The firing rate of the auditory nerve rapidly increases to the maximum at the onset of sustained stimulation followed by a gradual decay in firing rate (i.e., neural adaptation); neural activity and responsiveness to subsequent stimulation are reduced for a brief period following the cessation of the initial stimulation, resulting in forward masking effects (e.g., Smith, 1977). Neural adaptation plays important roles in speech encoding at the level of the auditory nerve (Delgutte, 1997). Fast neural adaptation and recovery from prior stimulation have been proposed to be important for producing peaks in the discharge rate of the auditory nerve that serve to enhance acoustic onsets in the speech waveform (Delgutte, 1997). Abnormal neural adaptation patterns, excessive adaptation and/or slow recovery from adaptation could potentially cause poor representation of temporal envelopes at the auditory nerve (Jeng et al., 2009), and might contribute to poor speech perception in some CI users (Wilson et al., 1994; Nelson and Donaldson, 2002).

**Figure 7:**
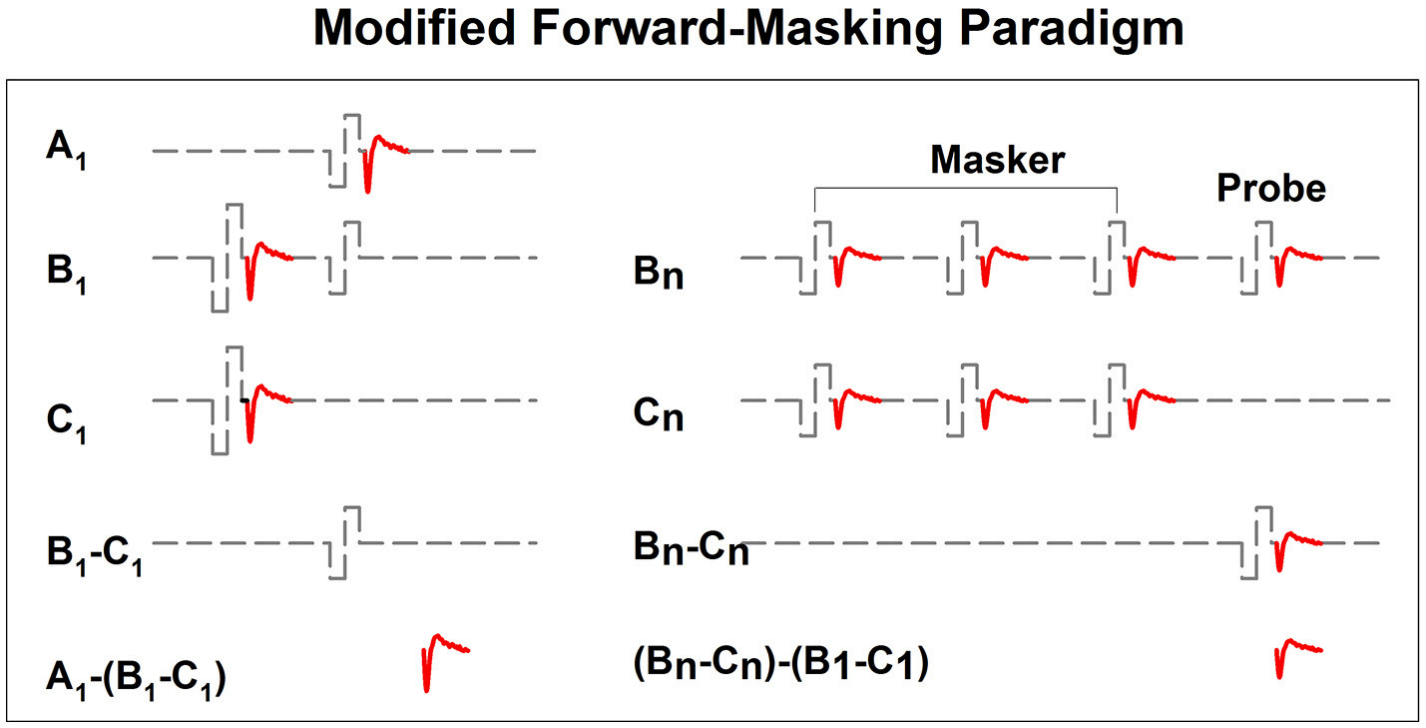
A schematic illustration of the modified forward-masking paradigm that can be used to measure eCAPs evoked by individual pulses in a pulse train. Gray dashed lines and red solid lines indicate biphasic electrical pulses and eCAP responses, respectively.

In implanted patients, neural adaptation of the auditory nerve can be evaluated by measuring eCAP amplitudes in response to individual pulses in a constant-amplitude pulse train using a modified forward-masking paradigm (Brown et al., 1990; Finley et al., 1997; Wilson et al., 1997; Rubinstein et al., 1999; Miller et al., 2000; Hay-McCutcheon et al., 2005; Hughes et al., 2012, 2014; McKay et al., 2013; He et al., 2016a). Figure 7 shows a schematic illustration of this paradigm. The left side of Figure 7 illustrates the classic two-pulse forward-masking paradigm (Brown et al., 1990). Subtracting trace C from trace B yields a template of the probe artifact. To derive eCAPs to each of the other pulses in a pulse train, a modification of the forward-masking technique is needed and shown schematically on the right side of Figure 7. In this paradigm, the MPI is adjusted to correspond to the period of the pulse rate minus the duration of one biphasic pulse. For example, the MPI is 1,943 μs if the pulse rate is 500 pps (period = 2,000 μs) and the pulse duration is 57 μs. With this increased MPI duration, coupled with the constant level pulses, some neural response is expected to be evoked by each successive pulse due to partial recovery from refractoriness. In an iterative process, the number of pulses comprising the masker is increased by one, with the final pulse in the pulse train always designated as the probe. For each iteration, the response to the final probe pulse is derived as (Bn-Cn)-(B1-C1), as shown on the right panel of Figure 7. One caveat is that the success of this method depends on one underlying assumption: the probe artifact stays constant during pulse train stimulation. However, this assumption may be invalid in some cases (He et al., 2016a; Tejani et al., 2017), which results in incomplete artifact removal. A careful inspection of derived eCAP waveforms is highly recommended for any study using this stimulation paradigm in order to identify cases where residual artifact exists. Unfortunately, there is still no method that can be used to overcome this technical challenge.

**Figure 8:**
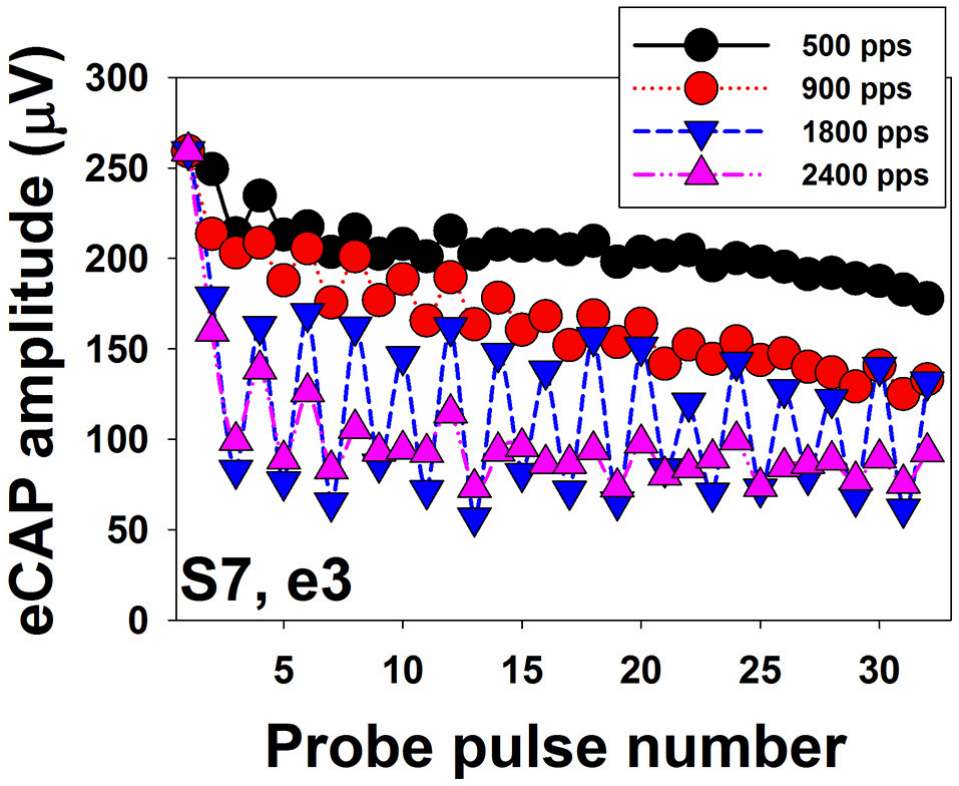
Amplitudes of eCAPs measured for individual pulses in a train of 32 pulses. Results measured at different pulse rates are indicated using different symbols and colors.

Figure 8 shows eCAP amplitudes in response to individual pulses of a train of 32 pulses measured at electrode 3 in one implanted child with SNHL (S7). Results are shown for four pulse rates, ranging from 500 to 2,400 pps. These data show that eCAP amplitudes measured at 500 pps (black symbols) rapidly decrease in the first few milliseconds after stimulus onset followed by a more gradual decline. It should be noted that this decline in eCAP amplitude typically does not occur for pulse rates of 200 pps or lower (Wilson et al., 1997), which suggests that the excitability of auditory nerve fibers fully recovers in these conditions between any two pulsatile stimulations (Wilson et al., 1997; Matsuoka et al., 2000a). At 900 pps (red symbols), eCAP amplitudes as a function of pulse numbers starts to show an alternating response pattern, with eCAPs to odd-numbered pulses having larger amplitudes than those evoked by even-numbered pulses. This alternating pattern typically occurs at pulse rates of 400–2,400 pps (Wilson et al., 1997; Hughes et al., 2012) and is believed to be a result of the refractory properties of auditory neurons (Finley et al., 1997; Wilson et al., 1997; Matsuoka et al., 2000b; Abbas et al., 2001). Theoretically, all neurons in the electrical field generated by the first pulse are available for activation at the maximum excitability. While these neurons are in their refractory phase, they will be unresponsive or have reduced excitability to the second pulse if the time period between these pulses is less than 3 or 4 ms (i.e., refractory period). At the time of the third pulse, many of these neurons will now be sufficiently recovered to be excited by the third pulse. Consequently, eCAP amplitude to the third pulse will be larger than that to the second pulse. This recovery-refractory process occurs during the entire process of pulse-train stimulation, which results in this alternating pattern (Wilson et al., 1997). The alternation in eCAP amplitude becomes more robust at 1,800 pps (blue symbols) in this case, as evidenced by a larger difference in amplitude between eCAPs evoked by the odd- vs. even-numbered pulses. The rate at which the maximum alternation occurs is typically around 900–1,800 pps (Hughes et al., 2012; He et al., 2016a), which presumably “resonate” with the RRP of the stimulated auditory nerve fibers (Matsuoka et al., 2000a; Hughes et al., 2012). In addition to this simple alternating pattern, complex alternating patterns, ranging from triplet to sextuplets patterns (i.e., increase and decrease in amplitude repeated every three–six responses) have been described in some studies (Wilson et al., 1997; Hughes et al., 2012; He et al., 2016a). The underlying mechanism of the complex alternating pattern or its clinical association with CI outcomes or programming settings remains unknown. Further increases in stimulation rate to 2,400 pps (yellow symbols in Figure 8) diminish the alternating pattern of eCAP amplitude due to stochastic independence among auditory nerve fibers. This stochastic state is caused by the combined effects of incomplete refractory recovery, increased neural adaptation, and increased temporal jitter (Hay-McCutcheon et al., 2005; Mino and Rubinstein, 2006). The rate at which the stochastic state occurs is typically at 2,000 pps or higher (Wilson et al., 1997; Rubinstein et al., 1999; Hughes et al., 2012). Even though high pulse rates are initially recommended due to its capability of inducing a stochastic state in which “pseudo-spontaneous” neural discharges occur, inconsistent results have been reported in terms of whether high pulse rates are beneficial for speech perception in CI users (e.g., Fu and Shannon, 2000; Loizou et al., 2000; Vandali et al., 2000; Friesen et al., 2005; Weber et al., 2007). Despite well-reported basic properties of eCAPs evoked by pulse train stimuli, it still remains unknown whether/how these eCAP response patterns are associated with speech and language outcomes or whether they can be used to select the optimal programming rate for individual CI patients.

Data shown in Figure 8 clearly demonstrate that eCAP amplitude decreases as the pulse rate increases. The amount of reduction in eCAP amplitude (i.e., adaptation) can be quantified by comparing amplitudes of eCAPs elicited by pulses occurring later in the pulse train to eCAP amplitudes elicited by early pulses (Hay-McCutcheon et al., 2005; Hughes et al., 2012, 2014; Zhang et al., 2013; He et al., 2016a). Although several studies have used eCAPs to measure the amount of neural adaptation in human CI users (Finley et al., 1997; Wilson et al., 1997; Rubinstein et al., 1999; Hay-McCutcheon et al., 2005; Hughes et al., 2012, 2014; McKay et al., 2013; He et al., 2016a), comparing results among these studies is challenging due to differences in duration of pulse train (ranging from 13 to 50 ms), pulse rate tested (ranging from 250 to 5,000 pps), and the time point used to calculate the amount of neural adaptation. To date, the association between neural adaptation of the auditory nerve and auditory perception in human CI users has only been evaluated in one study (Zhang et al., 2013). In this study, Zhang and colleague measured the neural adaptation of the audtory neve induced by a 50-ms pulse train with a pulse rate of 1,000 pps at one electrode in 14 post-lingually deaf adult CI users. For each subject, they also measured behavioral gap detectoin threshold (GDT) and speech perception scores. Their results showed no assocation between the amount of neural adapation of the auditory nerve and GDTs or speech perception scores. However, these results need to be interpreted with caution since only one electrode site was tested for adaptation of the auditory nerve in each subject despite the fact that adaptation varies across stimulation sites within individual patients (Hughes et al., 2012; He et al., 2016a). In contrast, behavioral GDTs and speech perception were evaluated through the speech processor using sound-field presentation at relatively high stimulation levels. As a consequence, results of Zhang et al. (2013) did not provide direct evidence for the effect of adaptation of the auditory nerve on perceptual sensivitiy to temporal gaps or speech perception capabilities in CI users. To date, it remains unknown to what extent neural adaptation of the auditory nerve affects auditory temporal processing and speech perception capabilities in CI users. Further studies are warranted in order to fill in these gaps in knowledge.

**Figure 9:**
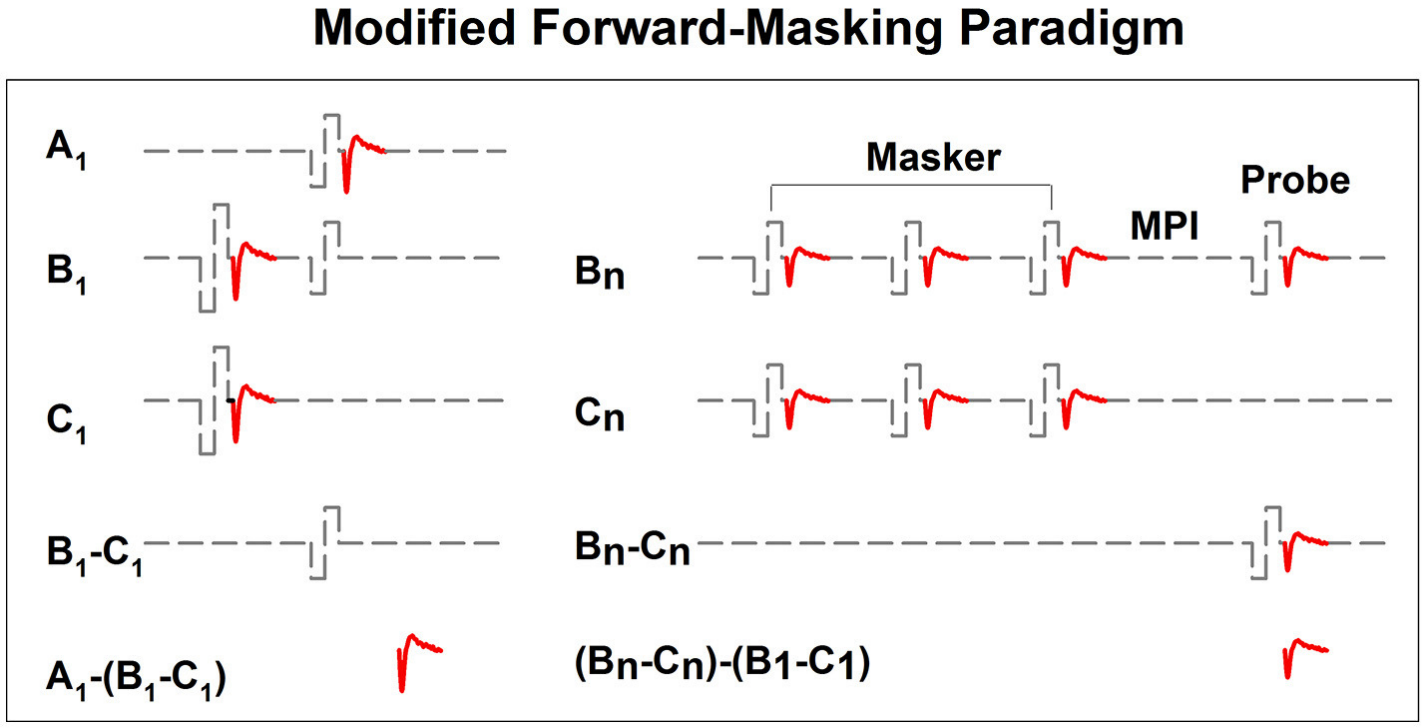
A schematic illustration of the modified forward-masking paradigm that can be used to evaluate recovery from neural adaptation introduced by a pulse train using eCAP recordings. Gray dashed lines and red solid lines indicate biphasic electrical pulses and eCAP responses, respectively.

Recovery from neural adaptation at the level of the auditory nerve can be evaluated by measuring eCAP amplitude in response to the probe pulse at different time points after the masker-pulse-train ceases. Two stimulation paradigms have been used for this purpose (Dhuldhoya, 2013; He et al., 2016b; Adel et al., 2017). A schematic illustration of the first paradigm is shown in Figure 9. This paradigm is very similar to the modified forward-masking paradigm shown in Figure 7 except for the varied MPI between the probe and the masker-pulse-train (right panel of Figure 9). As the MPI increases, the eCAP evoked by the probe pulse (i.e., [B'-C']-[B-C]) gradually recover from the neural adaptation induced by the masker-pulse-train. The adaptation recovery function (ARF) can be obtained by plotting eCAP amplitudes as a function of MPIs. In addition to this paradigm, the modified alternating polarity paradigm has recently been used to derive ARFs in human CI users. For details of this paradigm, please see Adel et al. (2017).

**Figure 10:**
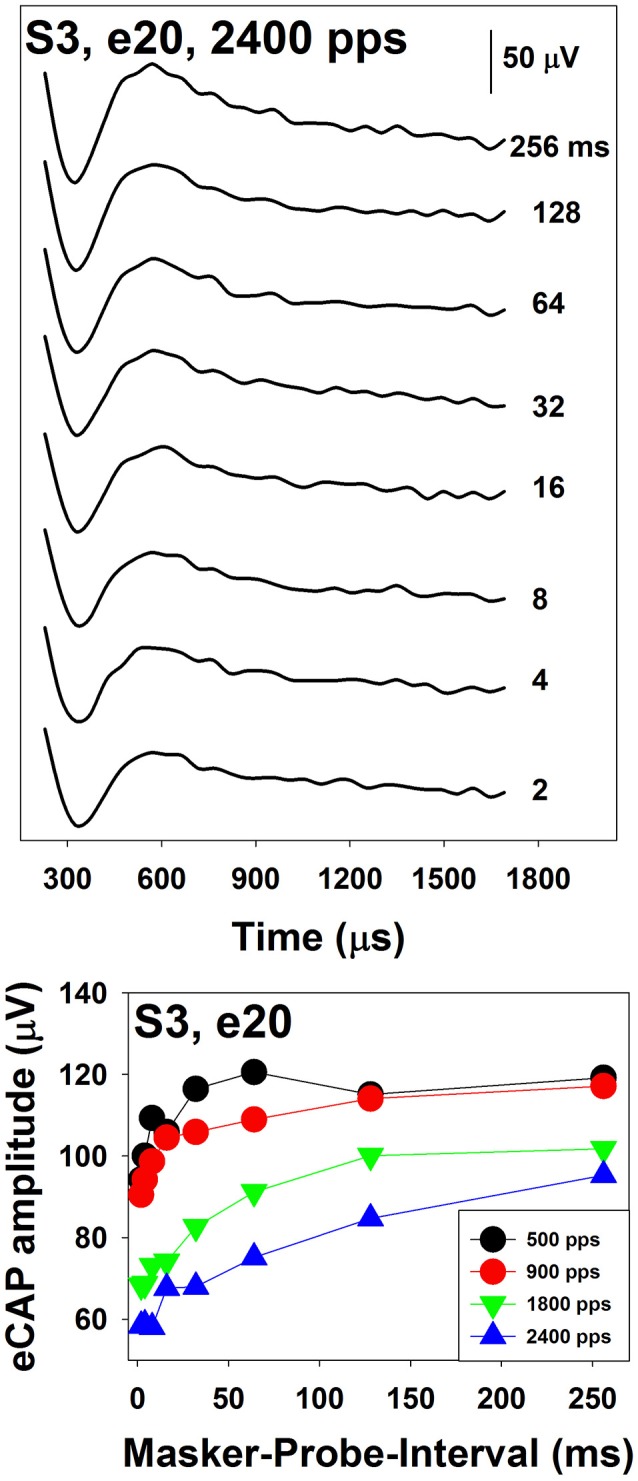
Neural adaptation recovery function measured at four pulse rates in S3. These results were measured for electrode 20 in a Cochlear CI user. Results recorded at different rates are indicated using different symbols.

The top panel of Figure 10 shows a series of eCAP waveforms measured at various MPIs at electrode 20 in S3. The masker was a 100-ms pulse train with a pulse rate of 2,400 pps presented at the C level. The MPIs used to measure these eCAPs ranged from 2 to 256 ms and are labeled for these traces. These data show that eCAP amplitudes are larger at longer MPIs. The bottom panel shows ARFs measured at four pulse rates ranging from 500 to 2,400 pps at the same electrode. These ARFs follow exponential distributions. eCAP amplitudes reach a plateau at longer MPIs for faster pulse rates, which suggests slower adaption recovery at faster pulse rates. As a result, ARFs measured at faster rates (green and blue symbols) appear to be flatter than those measured at slower rates (black and red symbols).

**Figure 11:**
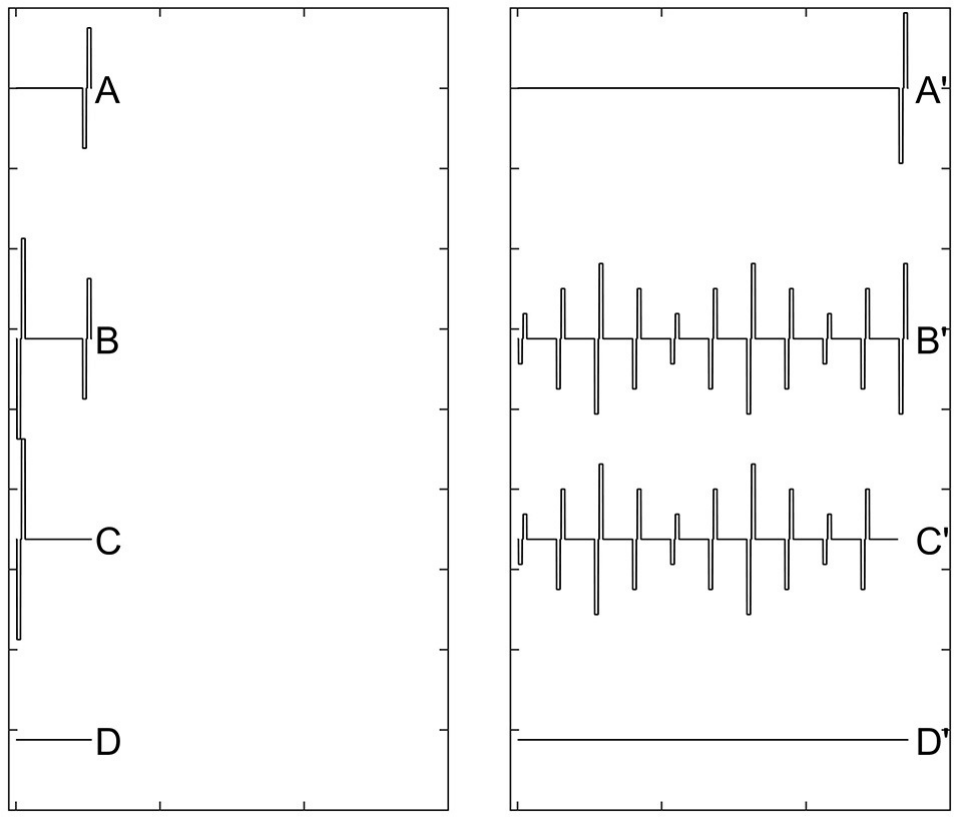
A schematic illustration of the stimulation paradigm that can be used to measure eCAPs evoked by individual pulses of a sinusoidally amplitude-modulated pulse train. This figure is courtesy of Viral D. Tejani at The University of Iowa.

The literature related to recovery from neural adaptation of the auditory nerve in CI users is relatively scarce. To date, only three studies have evaluated this specific issue (Dhuldhoya, 2013; He et al., 2016b; Adel et al., 2017). Overall, these studies showed that ARFs could consist of up to three components with an initial rapid increase (fast recovery) followed by a rapid decrease (adaptation enhancement) and a second slower increase (slow recovery) in eCAP amplitude (Dhuldhoya, 2013; He et al., 2016b). An example of the ARF with all three components is shown in Figure 11. In this example, the fast recovery is observed for MPIs of 1–2 ms, followed by the adaptation enhancement occurring at MPIs of 2–8 ms. The slow recovery is observed for MPIs of 16–256 ms. This example represents the most complicated ARF observed in human CI users. Not all reported ARFs have all three components. The slow recovery is the most commonly observed component in CI users (Dhuldhoya, 2013; He et al., 2016b). It has been proposed that the fast recovery is due to increased neural synchrony of auditory nerve fibers (Nourski et al., 2007), and the adaptation enhancement possibly results from the loss of current integration at the neural membrane due to long MPIs (Miller et al., 2011). The slow recovery is believed to reflect recovery from neural adaptation (Nourski et al., 2007; Miller et al., 2011). However, these interpretations may be oversimplified. High masker level or low probe level yields longer adaptation recovery in both adult and pediatric CI users (Dhuldhoya, 2013). At a fixed current level, increasing pulse rate yields long recovery from neural adaptation (He et al., 2016b; Adel et al., 2017). Preliminary data reported by He et al. (2016b) indicated that auditory nerve fibers in older CI users might have slower adaptation recovery than those of young CI patients. To date, our understanding of adaptation recovery of the electrically-stimulated auditory nerve in human listeners is still very limited. As a result, the potential clinical implication of the eCAP ARF is unclear.

#### Amplitude modulation encoding

**Figure 12:**
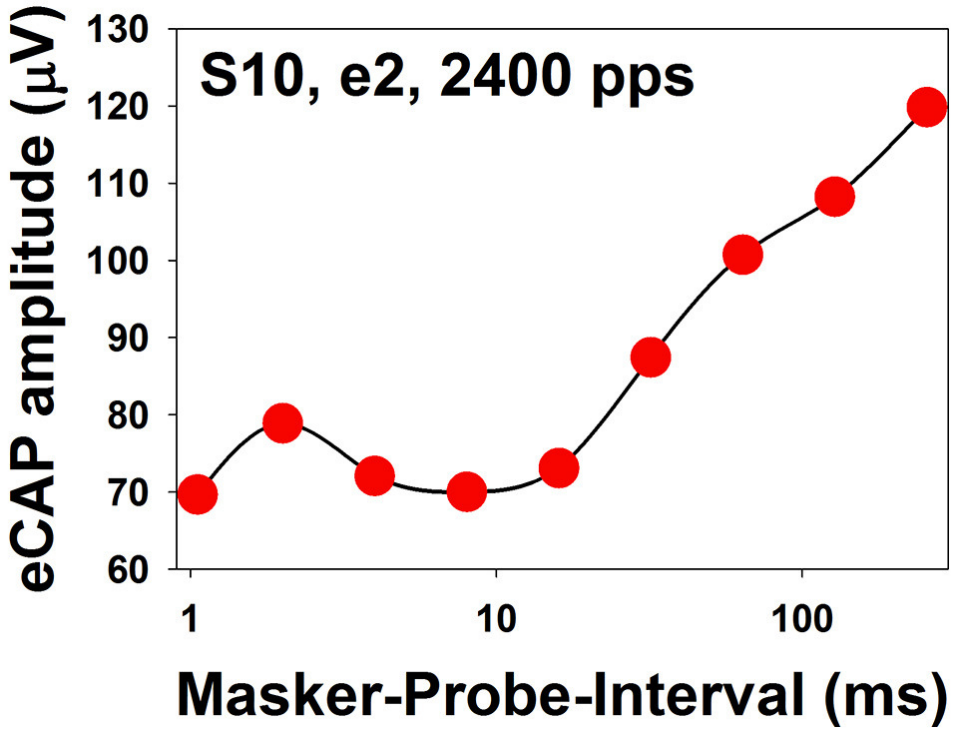
eCAP amplitudes measured at different MPIs for electrode 2 in one adult CI patient (S10). The stimulus was a 100-ms constant-amplitude pulse train with a pulse rate of 2,400 pps presented at the maximum comfortable level. MPIs are shown in a logarithmic scale.

Neural encoding of amplitude modulation cues at the level of the auditory nerve can be evaluated by measuring eCAPs evoked by individual pulses in an amplitude-modulated (AM) pulse train using a stimulation paradigm shown in Figure 12. This paradigm is the same as the modified forward-masking paradigm shown in Figure 7 with two important exceptions. First, the pulse train (right panel) is amplitude modulated. Second, the probe level used in the two-pulse forward masking paradigm (left panel) needs to be the same as that of the probe pulse in the AM pulse train (right panel). The eCAP evoked by individual pulses of the AM pulse train is derived by the subtraction of (B'-C')-(B-C).

**Figure 13:**
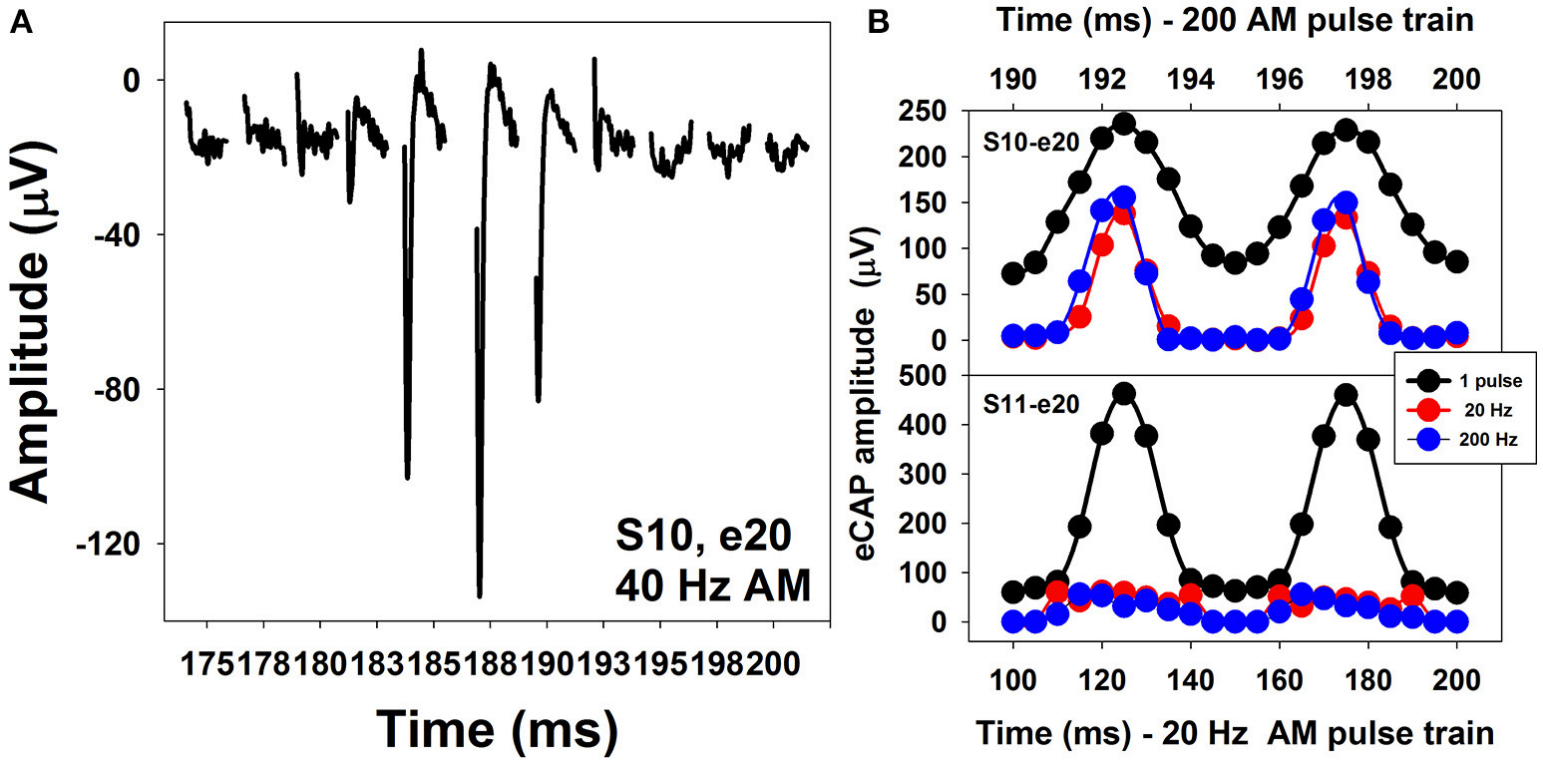
Waveforms of eCAPs recorded at electrode 20 in S10 **(A)** and eCAP amplitude modulation functions measured at electrode 20 in S10 and S11 **(B)**.

Figure 13A shows a series of eCAP waveforms evoked by a 200-ms pulse train with a carrier pulse rate of 2,000 pps that was sinusoidally amplitude modulated (SAM) at 40 Hz at electrode 20 (e20) in one adult CI user (S10). These eCAP recordings span one SAM cycle. These responses show a periodical change in amplitude, which tends to follow the SAM of the stimulus. Figure 13B shows amplitudes of eCAPs to pulse trains with SAM rate of 20 Hz (red symbols) and 200 Hz (blue symbols) plotted as a function of time measured at e20 in S10 and S11 (top and bottom, respectively). Both subjects are post-lingually deaf adult CI users. Amplitudes of eCAPs evoked by single pulses at each of the probe levels used in the AM pulse train are indicated in black. These results show that the auditory nerve near e20 in both subjects can robustly encode AM cues delivered by single-pulse stimulation. However, AM cues delivered by pulse-train stimulation are better transmitted by the auditory nerve in S10 than in S11 at both AM rates, as indicated by greater modulation depth of eCAP amplitudes measured in S10 than those recorded in S11. For both subjects, there is a phase shift (lead) in eCAP responses evoked by the pulse train relative to eCAPs evoked by the single pulse. These data are consistent with results reported in human CI users (Wilson et al., 1997; Tejani et al., 2017) and acutely deafened guinea pigs (Abbas et al., 1998; Jeng et al., 2009). This phase shift has been proposed to be due to non-linear growth of the eCAP amplitude and a combined effect of refractoriness, adaptation, and facilitation (Jeng et al., 2009).

The association between how the auditory nerve responds to AM stimuli and auditory perception in human CI users is the least understood feature among all topics covered in this review. Even though the feasibility of measuring eCAPs using SAM pulse trains has been established for almost 20 years (Wilson et al., 1997), this feature has only been investigated in human CI users in two studies (Carlyon and Deeks, 2015; Tejani et al., 2017). Carlyon and Deeks (2015) assessed the association between AM neural encoding as evaluated by eCAP measures and temporal pitch perception in CI users. Their results showed that the ability of the auditory nerve to faithfully encode and transmit AM cues might be important for pitch perception. Factors accounting for limitation of pulse-rate discrimination were beyond the auditory nerve. Tejani et al. (2017) evaluated how well the auditory nerve encoded SAM cues by measuring eCAPs in response to a SAM pulse train with a carrier rate of 4,000 pps and AM rates of 125, 250, 500, and 1,000 Hz in adult CI users. In addition, they examined the association between eCAP results and psychophysical measures of amplitude modulation detection threshold (AMDT) at these AM rates in these patients. Their results showed that amplitudes of eCAPs in response to SAM pulse trains reflected the overall periodicity of the stimuli. The amount of variation in eCAP amplitude correlated with AMDT at SAM rates up to 500 Hz, with larger variations associated with lower AMDTs. However, the association between results of eCAP and behavioral measures was not observed at the SAM rate of 1,000 Hz, which was proposed to indicate the limitation of central auditory encoding and processing of AM cues at high rates (Tejani et al., 2017). The extent of modulation in eCAP amplitude is affected by the modulation depth in stimulus and the electrode location (Carlyon and Deeks, 2015; Tejani et al., 2017). It has been shown that stronger modulations in eCAP amplitude are evoked by stimuli with larger modulation depths (Carlyon and Deeks, 2015; Tejani et al., 2017). At the fixed modulation depth, eCAPs recorded at the apical electrodes demonstrate stronger modulation in amplitude (Tejani et al., 2017).

### Neural survival

Due to the compromised functional status of the auditory system, hearing impaired patients presumably have less channels that provide useful information for auditory perception than normal-hearing listeners. The number of available “functional channels” should, in theory, associate with speech and language outcomes in CI patients. At the peripheral auditory system, the pattern and degree of neural survival of auditory fibers may be an important factor for the number of available “functional channels.” Developing tools for estimating the number of survival auditory fibers and predicting CI outcomes for individual patients has been a research topic for many years. There has been an increased interest in using the eCAP to estimate neural survival of auditory nerve fibers. However, a direct comparison between eCAP responses and spiral ganglion cell density in human listeners is not feasible. Therefore, animal models are used to identify eCAP measures that are sensitive to neural survivals (e.g., Miller et al., 1994; Prado-Guitierrez et al., 2006; Ramekers et al., 2014). These measures have been subsequently used in human CI users to evaluate their correlations with behavioral measures of auditory perception and/or speech perception (e.g., Brown et al., 1990; Gantz et al., 1994; Kim et al., 2010; Pfingst et al., 2015a; Schvartz-Leyzac and Pfingst, 2016). This section reviews studies related to one eCAP measure that has been studied for many years (i.e., slope of the eCAP I/O function) and the three most recently developed eCAP measures (sensitivity to inter-phase-gap, phase duration and pulse polarity).

#### Slope of the eCAP I/O function

In animal models, sleeper slopes of eCAP I/O functions have been found to be generally associated with higher spiral ganglion density (e.g., Miller et al., 1994; Pfingst et al., 2014, 2015a,b). However, the spiral ganglion density only accounted for 50% of the variance in the slope of eCAP I/O function (Pfingst et al., 2014). In human CI users, flatter slopes have been found to be associated with longer duration of hearing loss (e.g., Schvartz-Leyzac and Pfingst, 2016). Studies evaluating the association between the slope of eCAP I/O function and speech perception scores in human CI users show inconsistent results. Whereas, some studies reported better speech perception scores measured in CI users with sleeper slopes (Brown et al., 1990; Kim et al., 2010), other studies found no association between these two measures (Franck and Norton, 2001; Turner et al., 2002). Factors accounting for the inconsistency include, but are not limited to, relative small sample size, limited test electrode location, and heterogeneity of patients tested in these studies.

#### Inter-phase-gap and phase duration

In guinea pigs, sensitivity of the eCAP to changes in interphase gap (IPG) and phase duration (PD) of a biphasic pulse have been shown to be correlated with auditory nerve survival (Prado-Guitierrez et al., 2006; Ramekers et al., 2014). Results of these animal studies showed that increasing IPG and/or PD reduced threshold and increased amplitude of the eCAP, presumably due to current integration occurring at the cell membrane. Poor spiral ganglion survival reduces the magnitude of IPG and PD. To date, the effect of increasing IPG on eCAP responses in human CI users has been only examined in one study. Schvartz-Leyzac and Pfingst (2016) studied the effect of increasing IPG from 7 to 30 μs on eCAP amplitude and slope of I/O function in human CI users. Their results showed that increasing IPG generally yielded increased eCAP amplitude and steeper slopes of I/O function. However, this effect varied across subjects and electrode locations. It remains unknown whether variations in sensitivity to IPG affect auditory perception or CI outcomes. The effect of PD has not been investigated in human CI users.

#### Polarity sensitivity

The charge-balanced biphasic pulse used in current CI consists of a cathodic phase followed by an anodic phase. Both cathodic and anodic stimuli can generate spikes in auditory nerve fibers (e.g., van den Honert and Stypulkowski, 1984; Miller et al., 1998, 2004; Shepherd and Javel, 1999). Simulation results using biophysical models suggested that the site of spike generation differs for anodic and cathodic stimuli (Rubinstein, 1991; Rattay, 1999; Rattay et al., 2001; Joshi et al., 2017). In healthy auditory nerve fibers, both cathodic and anodic pulses activate peripheral processes to generate spikes at low stimulus level. At high stimulus level, the cathodic pulses still stimulate peripheral processes, whereas anodic stimuli inhibit peripheral processes and generate spikes at central axons. In cases where peripheral processes are absent or demyelinated, the only site that can be depolarized/activated by cathodic stimuli is the cell body (i.e., soma). Compared with the central axon, the soma has much higher threshold, which results in a higher cathodic threshold. In these cases, the excitability of the central axon to anodic stimuli at high stimulus levels is not affected. As a result, at an equal stimulus level, catholic-leading pulses are more effective at eliciting a neural response from intact human auditory nerve fibers, whereas anodic-leading pulses are more effective when peripheral processes are absent or demyelinated (Rattay, 1999; Rattay et al., 2001). Therefore, comparing the difference in eCAPs evoked by cathodic-leading vs anodic-leading pulses may provide useful information about neural survival of auditory nerve fibers (Undurraga et al., 2010).

**Figure 14:**
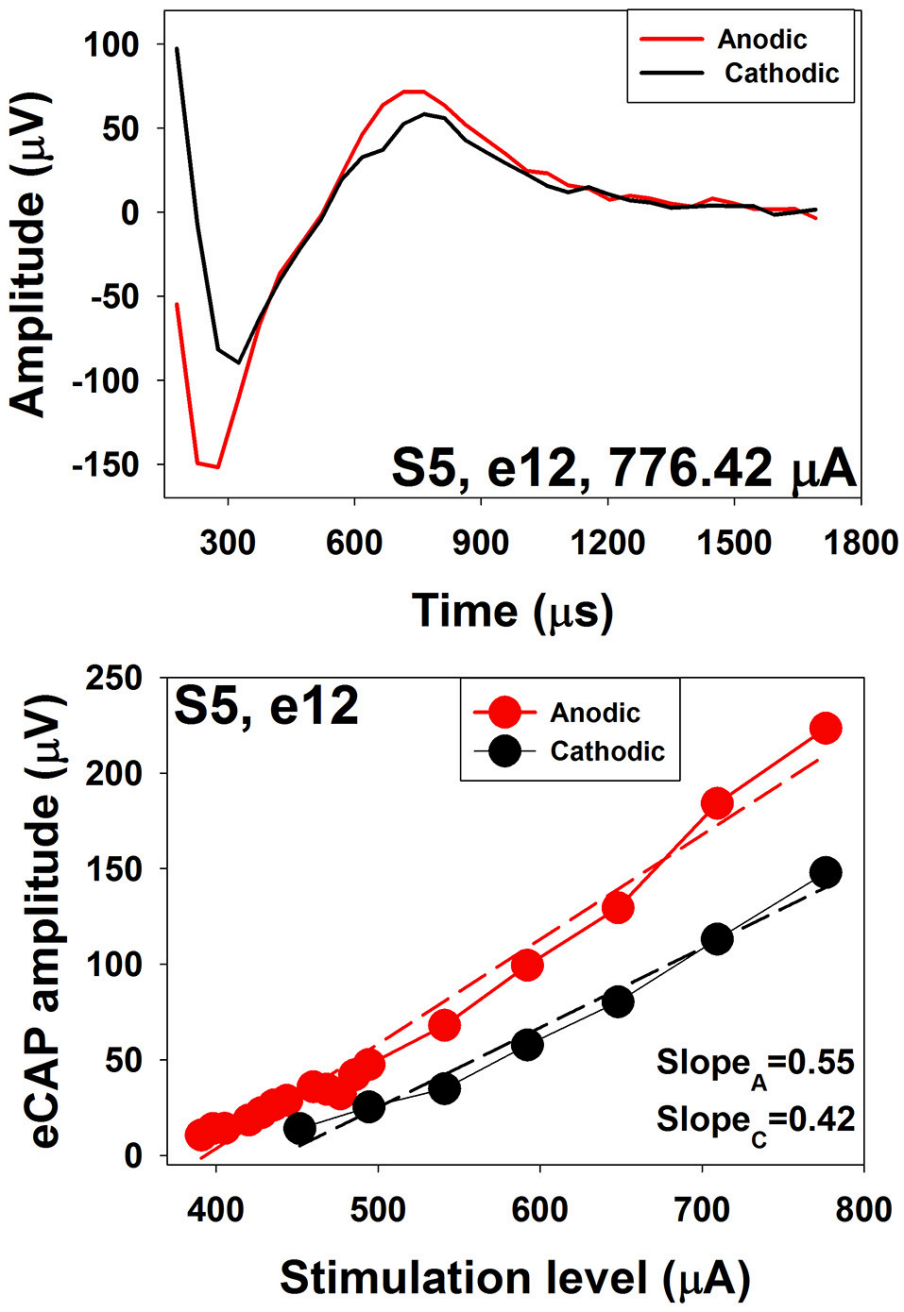
eCAP waveforms and I/O functions measured using stimuli with reversed polarities at electrode 12 in S5.

Several studies have investigated polarity sensitivity of auditory nerve fibers using eCAP recordings in human CI users (Macherey et al., 2008; Undurraga et al., 2010, 2012; Glickman et al., 2016). Results of these studies suggested that auditory nerve fibers in human CI users were more sensitive to the anodic phase than the cathodic phase of the phasic pulse. Specifically, at a fixed stimulus level, eCAPs evoked by anodic-leading biphasic pulses show larger amplitudes and shorter latencies than those evoked by cathodic-leading biphasic pulses (Macherey et al., 2008; Undurraga et al., 2010; Glickman et al., 2016). In addition, eCAP I/O functions measured for anodic-leading stimuli have lower thresholds and steeper slopes than those measured for cathodic leading pulses (Undurraga et al., 2010; Glickman et al., 2016). These results are consistent with the general belief that peripheral processes in deafened ears are demyelinated and degenerated (Fayad and Linthicum, 2006). The top panel of Figure 14 shows an eCAP evoked by an anodic-cathodic pulse (red line) and an eCAP evoked by a cathodic-anodic pulse (black line) measured at electrode 12 in one child Cochlear 24RE CI user. It is apparent that the eCAP evoked by the anodic-leading pulse has a larger amplitude and shorter latency than that evoked by the cathodic-leading pulse. The bottom panel shows eCAP I/O functions measured for both polarities. Dashed lines show results of linear regression fits. Slopes of these functions are indicated in the low right corner. These results demonstrate that the eCAP I/O function of the anodic-leading pulse (red symbols) has lower threshold and steeper slope than that measured for the cathodic-leading pulse (black symbols). Despite these exciting and promising findings, the association between speech perception capability and polarity sensitivity has not been evaluated in human CI user.

## Conclusions

This paper reviewed research efforts for investigating the utility of the eCAP in research and clinical practice, with an emphasis on new advances in knowledge and understanding that were gained within the last 10 years. Potential applications of the eCAP discussed in this paper include determining stimulus level, assessing spatial selectivity, evaluating temporal response properties and estimating neural survivals of auditory nerve fibers. It should be noted that substantial inter- and intra-subject variations across stimulating electrodes and/or pulse rates have been reported in all studies reviewed in this paper, which may reflect differences in the functional status of the neural populations that responded to electrical stimuli delivered by the CI. These variations highlight the importance of investigating to what extent differences in physioloigcal status of the auditory nerve can account for variations in auditory perception and speech perception across CI users and across stimulation sites within individual CI users. Despite these new exciting advances in our understanding of the eCAP, there are many questions that remain unknonwn. For example, it is unclear whether SOE functions measured using the eCAP can be used to determine which electrode should be used in programming MAPs for individual patients. In addition, the clinical and behavioral signfiance of different temporal response patterns of the auditory nerve remain unknown. Furthermore, whether difference in polarity sensitivity can be used to predict CI outcome for individual CI users remains unclear. These unknown questions provide exciting directions for future studies and leave room for developing new clinical applications for eCAP measures.

## Author contributions

SH designed and is accountable for all aspects of this study, as well as drafted and approved the final version of this paper. HT and CB participated in study design and are accountable for all aspects of this study. They also provided critical revision and approved the final version of this paper.

### Conflict of interest statement

The authors declare that the research was conducted in the absence of any commercial or financial relationships that could be construed as a potential conflict of interest.
